# Understanding CD30 biology and therapeutic targeting: a historical perspective providing insight into future directions

**DOI:** 10.1038/bcj.2017.85

**Published:** 2017-09-08

**Authors:** C A van der Weyden, S A Pileri, A L Feldman, J Whisstock, H M Prince

**Affiliations:** 1 Department of Haematology, Peter McCallum Cancer Centre, Melbourne, Victoria, Australia; 2 Haematopathology Unit, European Institute of Oncology, Milan, Italy; 3 Bologna University School of Medicine, Bologna, Italy; 4 Department of Laboratory Medicine and Pathology, Mayo Clinic, Rochester, MN, USA; 5 ARC Centre of Excellence in Advanced Molecular Imaging, Monash University, Clayton, Victoria, Australia; 6 Epworth Healthcare, Melbourne, Victoria, Australia; 7 Sir Peter MacCallum Department of Oncology, University of Melbourne, Parkville, Victoria, Australia

## Abstract

CD30 is a member of the tumor necrosis factor receptor superfamily. It is characteristically expressed in certain hematopoietic malignancies, including anaplastic large cell lymphoma and Hodgkin lymphoma, among others. The variable expression of CD30 on both normal and malignant lymphoid cells has focused research efforts on understanding the pathogenesis of CD30 upregulation, its contribution to lymphomagenesis through anti-apoptotic mechanisms, and its effect on cell survival. Given the restriction of CD30 to certain tumor types, the logical extension of this has been to attempt to exploit it as a therapeutic target. The efficacy of naked anti-CD30 antibodies in practice was, however, modest. Moreover, combinations with bacterial toxins and radioimmunoconjugates have also had limited success. The development of the antibody-drug compound brentuximab vedotin (BV), however, has rejuvenated interest in CD30 as a tumor target. Phase I and II clinical trials in Hodgkin lymphoma, peripheral T-cell lymphoma, cutaneous T cell lymphoma, and even CD30-expressing B-cell lymphomas, have shown the compound is well tolerated, but more importantly, able to deliver meaningful disease control even in patients with multiply relapsed or refractory disease. FDA approval has been granted for its use in relapsed Hodgkin lymphoma and systemic anaplastic large cell lymphoma. A recent phase III trial of BV in cutaneous T-cell lymphoma has confirmed its superiority to standard of care therapies. In this manuscript, we explore the history of CD30 as a tumor marker and as a therapeutic target, both in the laboratory and in the clinic, with a view to understanding future avenues for further study.

## CD30 molecule—cloning and characterization of tissue expression

CD30, also known as Ki-1or TNFRSF8, was first identified in 1982 using a monoclonal antibody (mAb) derived from a Hodgkin lymphoma (HL) cell line.^1, 2^ The CD30 molecule was subsequently cloned and characterized as a 120 kD transmembrane glycoprotein receptor belonging to the tumor necrosis factor receptor (TNFR) superfamily, with intracellular, trans-membrane and extracellular domains.^3, 4^ Sequence similarity between CD30 and other TNFR molecules is limited to the extracellular components; in CD30, these are comprised of six cysteine-rich repeats.^5^ Further delineation of CD30 epitopes suggested the extracellular components adopt a flower-like configuration.^6^ Later structural studies on other TNFR-related molecules—alone and in complex with ligands— suggest that the extracellular cysteine-rich repeats most likely adopt an extended conformation.^7^ This may have implications for antibody binding.

CD30 ligand (CD30L, also known as TNFSF8 or CD153) is a membrane-bound cytokine with sequence homology to other members of the tumor necrosis factor (TNF) family.^4^ CD30L can be detected *in vitro* on a subset of activated lymphocytes, histiocytes and granulocytes, and has been demonstrated on Reed–Sternberg cells and some T-cell lymphomas, although not consistently on anaplastic large cell lymphomas (ALCL).^8, 9, 10, 11, 12^ Additionally, an 88 kD form of soluble CD30 (sCD30) can be detected *in vivo* in inflammatory states and CD30-positive hematologic malignancies, and is presumed to represent a cleavage by-product of the extracellular portion of CD30.^13^

CD30 is expressed on a small subset of activated T and B lymphocytes, and a variety of lymphoid neoplasms, with the highest expression in classical HL and ALCL. It has been demonstrated with variable expression and intensity in some cases of peripheral T-cell lymphoma, not otherwise specified (PTCL-NOS); adult T-cell leukemia/lymphoma; cutaneous T-cell lymphoma (CTCL); extra-nodal NK-T-cell lymphoma; and a variety of B-cell non-HLs, including diffuse large B-cell lymphoma, particularly EBV-positive diffuse large B-cell lymphoma.^8, 14, 15, 16, 17, 18, 19, 20, 21, 22^ Neoplastic mast cells in advanced systemic mastocytosis have also been shown to be CD30-positive.^23, 24^ Less commonly, CD30 expression is seen in certain non-hematopoietic malignancies, including germ cell tumors and testicular embryonal carcinomas.^15^

The BerH2 antibody is used for routine assessment of CD30 expression in tissue specimens, with good correlation between immunohistochemical protein expression and specific mRNA levels.^25, 26^ Kim *et al.* reported the use of multi-spectral imaging in their study of brentuximab vedotin (BV) in CTCL, quantifying CD30 expression in biopsies otherwise designated CD30-negative by immunohistochemistry (IHC), thus defining a ‘low-level positive’ tumor group. Double antibody-conjugate techniques were employed to assess whether CD30 expression was representative of tumor, or of tumor-infiltrating inflammatory cells such as cytotoxic T-lymphocytes or macrophages.^27^ While these techniques are not widely available, these results raise interesting questions about the contribution of the tumor microenvironment to lymphomagenesis, the specificity of CD30 as a treatment target, and how to best assess patient suitability for anti-CD30 therapies.

While CD30-positivity in ALCL is defined as tumor cell expression of 75% or higher, diagnostic cut-offs for other tumor types have not been universally agreed upon. In mycosis fungoides (MF), for example, CD30 positivity is much less than in ALCL, with one group reporting median epidermal staining of 14% in non-transformed cases, with higher expression levels in more advanced stages and large-cell transformation.^28^ It is a matter of debate whether CD30 expression levels permit stratification of expected responses to anti-CD30 therapies.

## Role of CD30 in health and disease

Given the relative restriction of CD30 expression, early studies sought to characterize the driving factors behind CD30 upregulation in normal tissue. Initial studies showed that CD30 expression on lymphocytes could be induced by *in vitro* antigenic stimulation by mitogens or viruses, especially HIV, EBV and HTLV-1.^29^ Subsequent studies demonstrated that CD30 expression was not uniform across all activated lymphocytes, instead being limited to subpopulations of CD4^+^/CD45RO^+^and CD8^+^ T cells in lymph nodes and the thymic medulla. CD30 expression appears higher in CD4+ and CD8+ cells producing a Th2-type cytokine response,^4, 18, 30, 31, 32^ although subsequent studies have also demonstrated CD30 expression on Th0 and Th1-specific cells.^33^ Additionally, building on the work of Stein *et al.*, Catoretti *et al.* demonstrated that CD30 expression in B-lymphocytes is restricted to a minority population of stimulated B immunoblasts located at the edge of the germinal center and the extrafollicular region, with co-expression of markers of acute activation.^14, 34, 35^

CD30 knockout mice have been studied in an attempt to understand the role of CD30 expression, albeit with somewhat conflicting results. Amakawa *et al.* demonstrated that the development and maintenance of memory T cells, as well as T-helper cell-dependent B-cell class-switching, were not impaired in response to mitogenic stimulation in their CD30 mutant mice, which the researchers felt argued against CD30 being involved in maintenance of the immune response. The researchers additionally found that CD30 mutant mice had increased thymic volume and increased numbers of circulating double-antigen positive T cells, suggesting a role for CD30 in negative selection in the thymus, although this has not been confirmed by later researchers.^36^ Subsequent studies have, however, reported contrasting results to these earlier conclusions. Gaspal *et al.* demonstrated that secondary antibody production—a process dependent on follicular T cells—was impaired in mice deficient in CD30, with loss of a sustained germinal center response, even though the primary response to antigen stimulation was normal, a result recapitulated in CD30-OX40 co-deficient mice. These results were replicated by Kennedy *et al.*^37, 38^ Overall, these findings underscore a role for CD30 in immune surveillance and cross-talk between B and T cells.

**Figure 1:**
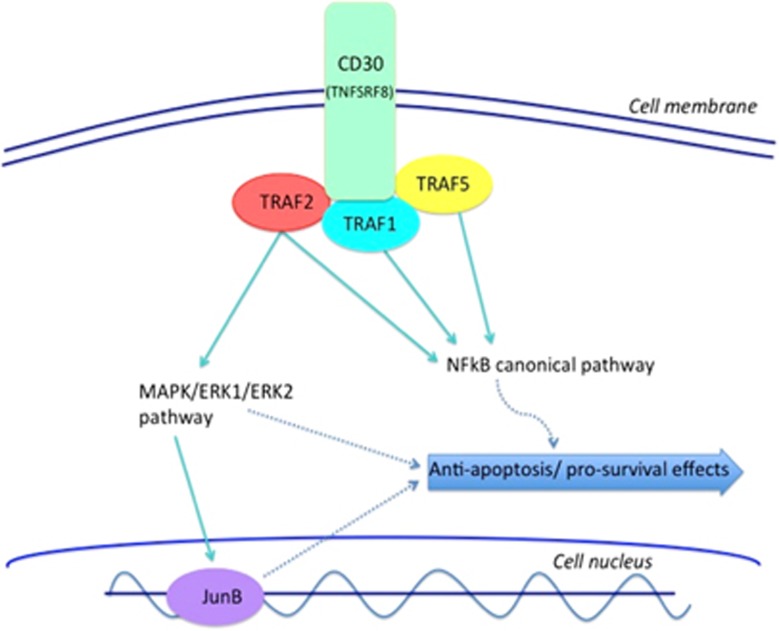
CD30 mediates its effects through a number of diverse signaling pathways, which in concert confer a survival benefit to the cells on which CD30 is upregulated. Stimulation of the CD30 molecule results in trimerization and signal mediation through tumor necrosis factor receptor-associated proteins (TRAF), in particular TRAF2, but also TRAF1 and TRAF5, to stimulate the nuclear factor-kappa B (NFkB) pathway. In addition to this, CD30 ligation also signals through the mitogen-activated protein kinase (MAPK) pathways, including ERK1 and ERK2, which have diverse anti-apoptotic and pro-survival benefits in the neoplastic cell. There appears to be a positive feedback loop between the MAPK/ERK pathway and the nuclear transcription factor JunB, which not only contributes to cell survival, but also upregulates CD30 expression.

Subsequent studies have assessed the effects of CD30 stimulation in order to understand the cell signaling pathways linked to CD30. Epitope stimulation of CD30 results in receptor trimerization and signal transduction via the recruitment of TNFR-associated factor (TRAF) and TRAF-binding proteins, generating a signaling complex.^5, 39^ TRAF2, as well as TRAF1 and TRAF5, have all been implicated in the signaling process. Downstream effects of CD30 stimulation are mediated in part by nuclear factor kappa B (NFkB), as well as by mitogen-activated protein kinase/extracellular signal-regulated kinase pathways, as outlined in Figure 1.^40, 41, 42, 43, 44^

The restricted expression of CD30 suggests the possibility that CD30 plays a role in the development and propagation of HL and ALCL. However, defining if and how CD30 *expression* relates specifically to lymphomagenesis—rather than being merely a marker of cell of origin—has proven challenging. Certainly the finding that the NFkB and mitogen-activated protein kinase/extracellular signal-regulated kinase pathways are integral to CD30-mediated signaling suggests that CD30 expression may confer a proliferative and anti-apoptotic benefit in neoplastic cells.^44, 45, 46^ Horie *et al.* proposed a link between CD30 overexpression and ligand-independent stimulation of the NFkB pathways in HL cells, underscoring a possible link between CD30 expression and tumor perpetuation. These findings were not replicated by Hirsch *et al.*, who instead suggested that NFkB activation in HL is constitutive and unrelated to CD30.^47, 48^ In contrast, Watanabe *et al.* showed that CD30 upregulation in HL and ALCL cell lines might be linked by a self-perpetuating loop through the mitogen-activated protein kinase/extracellular signal-regulated kinase pathway to the expression of JunB, a member of the activator protein (AP-1) transcription factor family, with diverse effects including a possible link to malignant transformation.^49^ Boddicker *et al.* demonstrated in ALCL cell lines that the transcription factor interferon regulatory factor-4 (IRF4) drives CD30 expression in a positive feedback loop involving NFkB.^50^ In addition to IRF4 and AP-1/Jun B, other groups have identified the Ets transcription family as being implicated in tumor cell CD30 upregulation.^50, 51, 52, 53^

Leading on from this, additional studies sought to define the role of CD30 *stimulation* in lymphoma pathogenesis; however, interpretation of the results is hampered somewhat by the use of differing ligands between studies. Early studies assessed the effects of the CD30 cognate, CD30L, in different cell lines. CD30L binding showed pleiotropic effects *in vitro*, with activation and enhanced cytokine secretion in HL cells, but a pro-apoptotic effect in ALCL cells.^54, 55^ Subsequent assessment of CD30 stimulation in ALCL and HL cell lines using the monoclonal antibody (mAb) Ki-1 also demonstrated a differential response, with decreased proliferation and increased apoptosis in ALCL cells, while HL cell lines were unaffected.^48, 56^ Gene expression profiling studies confirmed a marked difference in gene transcription between the HL and ALCL cell lines in response to CD30 stimulation.^48, 57^ Mir *et al.* suggested that the variable responses to CD30 stimulation in HL and ALCL cell lines might reflect the presence of a defective inhibitory protein in HL, permitting constitutive NFkB signaling, as well as insensitivity to CD30-mediated pro-apoptotic signaling.^56^

Later work by Buchan and Al-Shamkani suggested that the pleiotropic effects of CD30 stimulation might be mediated through the activation of different regions in the cytoplasmic tail. This raises the question of whether the activation of different regions is cell-type or ligand specific, and therefore whether this differential effect can be harnessed clinically.^45^ Indeed, one of the difficulties in understanding the pleiotropic effects of CD30 stimulation extends from the fact that these studies were performed in variable cognates in tumor cell lines, without a concomitant clear explication of the effects of CD30 stimulation by CD30L in normal tissues. More importantly, while CD30L can be detected in HL, it has not been as clearly demonstrated in ALCL, and as such, it is ultimately unclear whether it can be assumed to contribute to lymphomagenesis in all CD30-expressing tumors.

## CD30 as a tumor target

Given the overexpression of CD30 in certain lymphoma subtypes and some non-lymphoid neoplasms, it seems logical to exploit it as a therapeutic target.

### Monoclonal antibody monotherapy

#### Preclinical studies

The development of humanized anti-CD30 mAbs paved the way for clinical trials of immunotherapy. Initial preclinical studies in the mid- to late 1990s used murine anti-CD30 mAbs, with reported improved disease-free survival rates in xenograft mice treated with M44 or HeFi-1, and, in a later study using HeFi-1, tumor growth arrest or regression in an ALCL xenograft model.^58, 59^ Leading on from these studies, focus was directed to the creation of humanized mAbs, resulting in the development of SGN-30, a chimeric mouse-human antibody, and the fully humanized mAb 5F11 (MDX-060, or iratumumab).

Studies reported between 2002 and 2010 examined the effects of SGN-30 in HL and adult T-cell leukemia/lymphoma cell lines and mouse models. In contrast to earlier studies using non-humanized mAb, investigators were able to demonstrate cellular growth arrest and DNA fragmentation *in vitro* in HL cell lines treated with SGN-30. Moreover, tumor regression and improved survival was observed in a mouse xenograft cohort.^60, 61, 62^ One explanation advanced for this unexpected response in HL was that SGN-30 itself promotes receptor cross-linking and multimerization, potentially promoting growth arrest and pro-apoptotic signaling.^61^

Similarly, 5F11 was able to promote cell growth arrest in CD30-positive cell lines when receptor cross-linking occurred, enhancing antibody-dependent cell cytotoxicity. Tumor regression was seen in HL xenograft mouse models.^63^

#### Clinical studies

Based on promising results in preclinical studies, phase I and II trials of both SGN-30 and 5F11 were undertaken. However, the results of early phase studies did not seem to offer significant promise for the use of ‘naked’ anti-CD30 mAbs in practice.

The phase I trial of SGN-30 was conducted in 2002 and 2003, including 24 patients with relapsed or refractory HL or CD30-positive non-HL. SGN-30 was administered weekly for six doses, and was well tolerated. Clinical benefits were modest, with six patients achieving stable disease, and one patient with primary cutaneous ALCL achieving a complete response (CR).^64^

A subsequent phase II trial of SGN-30 in patients with HL and ALCL used the same dosing schedule with doses of either 6 mg/kg or 12 mg/kg; the regimen was again well tolerated. Clinical outcomes were, however, disappointing. Of the 38 patients with relapsed/refractory HL, 11 patients achieved stable disease, while none achieved CR or partial responses (PR). In the ALCL cohort of 41 patients, two CR and five PR were attained.^65^

Duvic *et al.* reported phase II results for the use of SGN-30 in relapsed/refractory cutaneous ALCL and other CD30-positive lymphoproliferative disorders. In this study, objective responses were seen in 16 of 23 patients (70%), with 10 patients achieving CR and six achieving PR. Overall clinical benefit rate was 87%, and median duration of objective response was 84 days. There appeared to be a dose-response effect, with some patients achieving clinical responses at 12 mg/kg despite having minimal benefit at 4 mg/kg.^66^

5F11 (MDX-060), a fully humanized anti-CD30 mAb, was the focus of a phase I/II trial in HL and systemic ALCL. Twenty-one patients were treated in the phase I cohort and 51 in the phase II cohort; of these patients, 25 achieved stable disease, two achieved PR and four achieved CR, with a median duration of response of less than 6 months in all groups. The investigators noted high rates of corticosteroid use in this study population, with 31 patients receiving steroids during treatment, including four patients with objective responses.^67^ Further clinical trials have not been pursued.

### Immunoconjugates

#### Immunotoxins

Early studies of anti-CD30 immunoconjugates focused on immunotoxins, albeit with limited success. The BerH2-saporin anti-CD30 immunotoxin used a ribosome-inactivating protein type 1 (RIP-1), with demonstrable efficacy *in-vitro* and in animal studies.^68, 69^ Later combinations included conjugates of BerH2 and other RIP-1s, the anti CD30-Pseudomonas exotoxin A conjugate Ki-4 (scFv)-ETA', and the anti-CD30 ricin A-chain immunotoxin.^70, 71, 72, 73^ However, the efficacy of these immunotoxins in humans was hampered by high rates of the development of anti-therapeutic antibodies, CD30 downregulation, or non-specific binding of the immunotoxin to soluble CD30.^71, 74^

#### Radioimmunoconjugates

Anti-CD30 radioimmunoconjugates have shown mixed results in preclinical studies, with limited scope for clinical development. A conjugate of Ki-4 and radiolabeled iodine (I-131) was tested in 22 patients with HL, with one CR and five PR, but had significant side effects, with seven patients experiencing grade 4 hematologic toxicity.^75^

Later combinations using Ki-4 and 5F11 antibodies with variable iodination showed high *in vitro* tumor cell specificity, but minimal efficacy *in vivo* in mouse models.^76^ The combination of HeFi-1 with either radiolabeled astatine (At-211) or yttrium (Y-90) showed preliminary efficacy in murine models; however, no clinical trials eventuated.^77^

#### Brentuximab vedotin

The development of a novel antibody-drug conjugate (ADC), BV (also referred to as SGN-35 or cAC10-vcMMAE), seemed to overcome some of the problems seen with SGN-30. Brentuximab vedotin consists of the humanized IgG_1_ mAb SGN-30, in combination with the antimitotic agent monomethylauristatin E (MMAE), joined by a cathepsin cleavable linker (valine-citrulline).^78^

Brentuximab vedotin acts through binding of the ADC to CD30-positive cells, followed by receptor endocytosis and release of MMAE upon exposure to intracellular lysozymes. This results in inhibition of tubulin formation and cell apoptosis. Bystander cells may also be susceptible to effluxed MMAE, with at least one study demonstrating apoptotic induction in co-cultured CD30-negative tumor cells.^79, 80^ Additionally, it seems that MMAE itself promotes T-cell expansion and activation, not only through enhanced antigen presentation, but also via a direct priming effect on dendritic cell maturation and differentiation.^81^

#### Brentuximab vedotin—preclinical studies

Preclinical studies confirmed efficacy of BV *in vitro* and in mouse models. Francisco *et al.* used BV in HL and ALCL cell lines, demonstrating *in vitro* stability, and ADC selectivity for CD30-positive cells. More importantly, BV successfully induced cell cycle arrest and apoptosis. In xenograft mouse models, BV treatment resulted in partial tumor regression in an HL model and complete tumor regression in an ALCL model, with a significant difference in tumor responses when compared to the ‘naked’ antibody. These treatment responses held true in mouse models of both subcutaneous and disseminated ALCL.^78^

Following on from this, Maeda *et al.* demonstrated a growth inhibitory effect of both SGN-30 and SGN-35 in adult T-cell leukemia/lymphoma cell lines. More importantly, mouse models treated with SGN-35 showed tumor regression.^60^

Blatt *et al.* examined the effects of BV in CD30-positive systemic mastocytosis. The researchers confirmed CD30 expression in systemic mastocytosis cell lines, and demonstrated cell apoptosis and death in response to BV. Interestingly, BV exposure did not appear to increase the risk of histamine release, and, moreover, appeared to downregulate IgE-mediated manifestations of histamine release.^23^

#### Brentuximab vedotin—clinical studies

Based on the promising results of preclinical studies, a phase I trial of BV was conducted in 45 patients with relapsed/refractory CD30-positive hematologic malignancies, including 42 patients with HL, two with systemic ALCL, and one with angioimmunoblastic T-cell lymphoma. The median number of prior therapies in this group was three (range one to seven), including 33 patients (73%) who had undergone an autologous stem cell transplant (auSCT); 17 patients had objective responses, including 11 CR, with a median duration of response of 9.7 months. Tumor regression was seen in 36 of 42 patients (86%) evaluable for radiologic response. The main side effects were fatigue, nausea, diarrhea, neutropenia, and peripheral neuropathy, with the latter seen in 16 patients. The recommended phase 2 dose was 1.8 mg/kg.^82^

Phase II and III studies using BV followed thereafter, as summarized in Table 1.

Pro *et al.* reported results for a cohort with relapsed/refractory systemic ALCL. 58 patients were dosed with 1.8 mg/kg every three weeks; 50 patients (86%) achieved objective responses, including 33 patients attaining CR and 17 patients attaining PR. The median duration of overall response was 12.6 months, and the median CR duration was 13.2 months. The most common grade 3 or 4 side effects included neutropenia, thrombocytopenia, and sensory neuropathy in 12% of patients.^83^

Younes *et al.* reported results for 102 patients with HL who had relapsed after auSCT. The objective response rate (ORR) was 75%, with a CR rate of 34%. Median duration of response was 6.7 months in all responders, and 20.5 months in those patients achieving CR. These findings are important given that the median number of prior treatments excluding auSCT was three, with a median time to relapse after transplant of 6.7 months, suggesting relatively chemo-refractory disease. Side effects were similar to those reported by Pro *et al.*; twenty patients discontinued therapy due to adverse events, predominantly sensory or motor neuropathies.^84^ A smaller study by Horwitz *et al.* in 35 patients with PTCL showed an ORR of 41%, with eight patients achieving CR. While the median progression-free survival (PFS) was 6.7 months in patients with angioimmunoblastic T-cell lymphoma, median PFS was a disappointing 1.6 months in the PTCL-NOS subgroup.^85^

In CTCL, phase II trials have been conducted in patients with Sezary syndrome and mycosis fungoides (MF), as well as in primary cutaneous ALCL and lymphomatoid papulosis. Kim *et al.* reported objective responses in 21 of 30 patients with either Sezary syndrome or MF, with seven patients having >90% clearance of cutaneous disease. It is notable that clinical response was not limited to those patients with >10% CD30 expression by IHC.^27^ Duvic *et al.* reported an ORR of 73% in their cohort of 48 patients, including CR in 17 patients. Mirroring the findings of Kim *et al.*, responses were seen even in MF/Sezary syndrome patients with low CD30 expression, defined as <10% by IHC. All 11 patients with either primary cutaneous ALCL or lymphomatoid papulosis responded to BV; however, the median duration of response in this group was only 26 weeks.^86^

Prince *et al.* have recently reported the results of a phase III trial in CTCL of BV versus physician’s choice of either methotrexate or bexarotene. This study of 128 patients with CTCL included 97 patients with MF and 31 with primary cutaneous ALCL. The cut-off for CD30 positivity in this study was defined as 10% in at least one tumor sample; multiple biopsies could be submitted for central review, and not all biopsies had to meet this cut-off requirement. Primary endpoints were overall response rates of four months or longer (ORR4) and PFS. Both ORR4 and PFS strongly favored BV, with ORR4 of 56 versus 13% (*P*<0.0001) and median PFS of 16.7 versus 3.5 months (HR 0.27, *P*<0.0001), with a median follow-up of 17.5 months.^87^

Expanding the treatment spectrum, Jacobsen *et al.* reported an ORR of 44% in a phase II trial of BV in relapsed refractory B-cell lymphoma, including a majority cohort with diffuse large B-cell lymphoma (49 of 68 patients). Eight patients attained CR, with median response duration of 16.6 months. Median PFS was four months (0.6+ – 24+ months).^21^ Bartlett *et al.* reported on the use of BV in a cohort of 52 diffuse large B-cell lymphoma patients designated CD30 negative with conventional IHC, with an ORR of 31% and CR rate of 12%. With computer-assisted digital image analysis, 11 of 16 responders were designated as low level positive for CD30 (CD30⩾1%).^88^

#### Brentuximab vedotin—additional treatment options

Bartlett *et al.* demonstrated that retreatment is feasible, treating 21 patients with HL and eight with ALCL who had previously achieved at least PR to BV. Overall response rate was 60% in the HL cohort and 88% in the ALCL cohort, with CR rates of 30 and 63%, respectively. As expected, sensory and motor neuropathy rates were higher than in upfront treatment, but the regimen was otherwise well tolerated.^89^

As with other antibody therapies, the next logical step was to use BV in combination. The use of BV with ABVD or AVD for upfront treatment of HL demonstrated significant additive pulmonary toxicity in the bleomycin-exposed cohort. Subsequent withdrawal of the bleomycin from the combination regimen did not appear to compromise efficacy.^90^ The phase III trial comparing ABVD and AVD with BV has completed recruitment; however, final results are pending. Promising results have been reported in a phase I trial in CD30-positive PTCL, using either BV administered sequentially with CHOP, or BV in combination with CHP. ORR was 85% in the sequential treatment arm, and 100% in the combination arm, with CR rates of 62 and 88%, respectively.^91^ Additionally, early reports of a phase I/II trial of BV and bendamustine in patients with relapsed HL or ALCL suggest the combination is well tolerated, with an ORR of 67%.^92^

Other treatment options include the use of BV as consolidation rather than salvage. Moskowitz *et al.* reported on their double-blind, randomized controlled trial assessing BV consolidation after auSCT for patients with relapsed HL. There was a significant improvement in risk of progression in the BV-treated cohort, with a median PFS of 42.9 months compared to 24.9 months in the placebo-treated cohort. By independent review, the estimated 2-year PFS was 63% in the BV cohort and 51%in the placebo group. There was, however, no significant difference in overall survival between BV and placebo arms.^93^

The results of these studies raise some interesting questions, one of which is whether the efficacy of BV lies solely in its capacity to affect directed cell death. This does not seem to be the case, with evidence of enhanced T-cell activation as well as dendritic cell priming and maturation in mouse models treated with microtubule-depolymerizing agents akin to that in BV.^81^ In this context, the combination of BV with immune checkpoint inhibitors is a rational one, and indeed, has been the focus of a recent phase I/II trial. The other question is whether the predicted efficacy of BV can be stratified according to the level of CD30 positivity—and consequently, given the observed responses in patients nominally designated as low-level positive or even negative by IHC techniques, whether we need to reassess the way in which CD30 positivity is defined in the laboratory.

#### Brentuximab vedotin—side effects and resistance

While BV is undoubtedly more effective than naked mAbs and other conjugate therapies, it does not entirely overcome the problems seen with earlier immunotoxin compounds. Despite the documented cellular specificity of BV, the recognition of dose-limiting and cumulative peripheral neuropathy with treatment suggests the compound has off-target effects. This is likely mediated in part through the anti-tubulin effects of MMAE.^79^ Hansen *et al.* have recently shown that HL cells can release CD30-containing extracellular vesicles. These vesicles then bind to CD30L-expressing cells in the tumor microenvironment, subsequently causing off-target binding of SGN-35 *in vitro*.^94^

In addition to the side effects reported in the major studies, subsequent case series have highlighted some concerning BV toxicities. Cases of pancreatitis—fatal at least one instance—have been reported.^95^ Progressive multifocal leukoencephalopathy, an incurable and often fatal CNS infection, has also been reported associated with BV.^96^

Resistance can be an issue with repeated BV exposure. Chen *et al.* have demonstrated diverse resistance mechanisms in HL and ALCL cell lines, including increased expression of drug transporter proteins, and MMAE resistance. While CD30 downregulation is a putative mechanism, and was seen *in vitro*, this finding was not seen in the tissue samples examined in this study,^97^ mirroring prior case reports of persistent CD30 expression even in the setting of reduced BV efficacy.^98^ However, cases of relapsed/refractory ALCL with downregulation of CD30 following BV treatment have also been reported.^99, 100, 101^

### Other therapeutic approaches

Phase I and II trials have been performed using bi-specific molecules incorporating anti-CD30 mAbs; however, the utility of these molecules has been hampered by the invariable development of antibodies against the agents. One such molecule combined an anti-CD30 mAb with the bi-specific mAb HRS-3/A9, directed against CD16, aiming to enhance recruitment of natural killer cells and phagocytic cells. A phase I/II clinical trial was conducted in 15 patients with relapsed/ refractory HL, with one CR and one PR lasting six and three months respectively. Nine of the 15 patients developed antibodies to the bi-specific molecule, leading to treatment discontinuation.^102^

Similarly, an anti-CD64/anti-CD30 molecule H22 x Ki-4 was developed in the late 1990s. This combined the antigen-binding region of Ki-4 with a humanized CD64-specific mAb, with the theoretical rationale being that CD64, as part of the high affinity immunoglobulin receptor, should enhance antibody-dependent cell-mediated cytotoxicity. While it was well-tolerated in a phase I trial, with objective responses in four of 10 patients with multiply relapsed/ refractory HL, interest in these agents has waned.^103^

The most recent efforts in targeting CD30 have focused on the use of chimeric antigen receptor T (CAR T) cells. Hombach *et al.* reported on the safety of CD30-specific CAR T therapy, demonstrating in mouse models that CD30/CD34-positive normal hematopoietic cells and activated lymphocytes were not targeted by CD30 CAR T cells.^104^ More importantly, Wang *et al.* have recently published preliminary results for a phase I trial, showing safety and efficacy, with PR in seven of the 18 patients treated, and stable disease in six.^105^

## Future directions

We are yet to fully define the significance of tumoral CD30 expression, that is, whether the molecule merely signifies cell of origin, for example, ‘stimulated lymphocyte’, whether its expression plays some role in perpetuating a malignant phenotype, or whether it reflects the recruitment of an inflammatory milieu that enhances tumor growth and survival. Indeed, CD30 expression may reflect a combination of all of these possibilities, and the relative balance of each may inform our thinking about tumor pathogenesis as well as how best to target CD30 therapeutically. Certainly it seems that merely demonstrating CD30 expression does not guarantee responses to anti-CD30 therapies, as demonstrated by the disappointing results of BV in PTCL-NOS, and the recently reported failure to achieve the ORR endpoint in a phase II study of BV in primary mediastinal B-cell lymphoma, despite strong CD30 expression in this tumor type.^106^ Conversely, the efficacy of BV in some tumors even with low-level CD30 expression is intriguing, suggestive of off-target immune modulatory effects of the compound, as discussed earlier.

The pleiotropic responses to CD30 stimulation are additionally of interest. Some of the studies outlined earlier would suggest the variable responses reflect differing mechanisms by which CD30 is upregulated in different tumor types, with consequent diverse effects on the signaling pathways mediated through CD30. The unanswered question is whether varying the type or mechanism of CD30 ligand binding may allow us to specifically target pro-apoptotic cellular signaling pathways, or to counteract cellular survival mechanisms. The finding that apoptosis may be augmented with antibody cross-linkage suggests this might be the case; however, this has not been studied in depth.

From a therapeutic standpoint, the failure to achieve substantive benefits with monoclonal antibodies and other antibody–drug combinations is disappointing. It is clear that BV overcomes many of these issues; however, the neurotoxicity of the MMAE may limit its long-term use. This raises the issue of why ‘naked’ antibody therapies are not as successful as expected. Some possibilities have been addressed, for example, it does not appear to be due to an excess of antibody binding to soluble CD30.^107^ The paucity of available structural data limits our understanding of the way in which CD30-antibody interactions occur, such as the possibility that the interaction between CD30 and anti-CD30 antibodies may orient the antibody in a way that is unable to properly induce antibody-dependent cell-mediated cytotoxicity. X-ray or cryo-electron microscopy assessment of the structure of CD30 alone, CD30 in complex with CD30L, and CD30 bound to SGN-30 and SGN-35, would be invaluable in investigating these possibilities.

It has additionally been suggested that the differential response to anti-CD30 antibodies in HL and ALCL may in fact reflect a pro-survival benefit conferred by, and specific to, the HL microenvironment.^64, 108, 109^ Supporting this postulate is the observation of a ‘bystander’ effect on surrounding, non-CD30-positive cells exposed to BV, which may alter the microenvironment immune signaling in HL.^81, 94^ Clinical trials of the combination of nivolumab and BV are currently under way, with preliminary results suggesting that the combination is safe and well-tolerated.^110^ Trials combining the naked antibody with immune-checkpoint inhibitors would be an interesting question for future study.

Some of the therapeutic approaches targeting CD30 currently under investigation in clinical trials are summarized in Table 2. These trials are focused predominantly around the use of BV in combination with other agents, or exploring other therapeutic niches for BV, such as salvage as a bridge to transplant. Of note, however, is the use of anti-CD30 chimeric antigen receptor T cells, the results of which are awaited with interest.

The limited expression of CD30 to certain tumor types remains a tantalizing target in an era of purpose-designed therapies. Historical attempts at targeting CD30 may have failed due to a lack of clear understanding of the mechanisms and significance of CD30 expression in both health and in disease. Answering the question of how to maximize an anti-CD30 strategy into the future may require us to return to the laboratory bench, using contemporary structural techniques, molecular profiling and immunological modeling.

## Figures and Tables

**Table 1:** Phase II and III trials of brentuximab vedotin (BV)

*Trial*	*Phase*	*Disease subgroup*	*Patient number*	*Median age in years (range)*	*Response rates*	*Response duration*	*Survival*
Pro *et al.*^83^	II	sALCL	58	52 (14–76)	ORR 86% (95% CI 74.6–93.9) CR 57% (95% CI 43.2–69.8) PR 29%	Median DOR 12.6 months (95% CI 5.7–NE)	Median PFS 13.3 months (95% CI 6.9–NE) Median OS not reached
Younes *et al.*^84^	II	HL	102	31 (15–77)	ORR 75% (95% CI 64.9%–82.6%) CR 34% (95% CI 25.2%–44.4%) PR 40%	Median DOR 6.7 months (95% CI 3.6–14.8)	Median PFS 5.6 months (95% CI 5.0–9.0) Median OS 22.4 months (95% CI 21.7–NE)
Horwitz *et al.*^85^	II	PTCL	35	64 (33–83)	ORR 41% (95% CI 24.6–59.3) CR 24% PR 18%	Median DOR 7.6 months (95% CI 1.3–14+)	Median PFS 2.6 months Median PFS in AITL cohort 6.7 months Median PFS in PTCL-NOS cohort 1.6 months Median OS not reported
Duvic *et al.*^86^	II	CTCL (MF, pcALCL, LyP)	48 (28 with MF, 9 with LyP, 2 with pcALCL)	59.5 (31–77)	ORR 73% ORR 54% in MF group ORR 100% in other subgroups CR 2/28, PR 13/28 in MF subgroup	Not reported	PFS 1.1 years (95% CI 0.9–1.4)
Kim *et al.*^27^	II	CTCL (MF and SS)	32 (30 evaluable for efficacy)	62 (20–87)	ORR in 21/30 (70%, 90% CI 53–83) CR in 1/30 PR in 20/30SD in 4/30	Not reported	Median PFS not reached at 12 months Median EFS >6 months 61% event free at 6 months 28% event free at 12 months
Jacobsen *et al.*^21^	II	B-cell lymphoma	68 (48 with DLBCL, 19 other B-cell lymphomas)	62 (17–85) in DLBCL cohort 36 (16–68) in other lymphoma cohort	ORR 44% in DLBCL cohort (95% CI 29.5–58.8) CR 17% PR 27% ORR 26% in other lymphoma cohort (95% CI 9.1–51.2) CR 16% PR 11%	Median DOR in DLBCL cohort 5.6 months (0–22.7+ months)	Median PFS in DLBCL cohort 4 months (0.6–24 months)
Prince *et al.*^87^	III *Comparison of BV with physician’s choice of either bexarotene or methotrexate*	CTCL	128 (97 with MF, 31 with pcALCL) with 64 in BV group, 64 in PC group	62 (22-83) in BV group 58 (22–83) in PC group	ORR4 56.3% (BV group) versus 12.5% (PC group), with *P*<0.0001 ORR 67% in BV group CR 16% ORR 20% in PC group CR 2%	Not reported	Median PFS 16.7 months (BV group) versus 3.5 months (PC group), with *P*<0.0001
Bartlett *et al.*^88^	II	DLBCL	52		ORR 31% CR 12%		Median PFS 1.4 months (0.4–15.6) Median OS 7.5 months (0.7–18.6+)

Abbreviations: AITL, angioimmunoblastic T-cell lymphoma; CI, confidence interval; CR, complete response; CTCL, cutaneous T-cell lymphoma; DLBCL, diffuse large B-cell lymphoma; DOR, duration of objective response; EFS, event-free survival; HL, Hodgkin lymphoma; LyP, lymphomatoid papulosis; MF, mycosis fungoides; NE, not evaluable; ORR, objective response rate; ORR4, objective response rate at 4 months; OS, overall survival; PC, physician’s choice; pcALCL, primary cutaneous anaplastic large cell lymphoma; PFS, progression-free survival; PR, partial response; PTCL, peripheral T-cell lymphoma; PTCL-NOS, peripheral T-cell lymphoma, not otherwise specified; sALCL, systemic anaplastic large cell lymphoma; SD, stable disease; SS, Sezary syndrome.

**Table 2:** Current clinical trials using CD30 as a therapeutic target

*Tumor subtype*	*Subgroup eligibility*	*Other criteria*	*Trial type*	*Intervention*
NHL *or* HL with CD30 expression	R/R		Phase Ib/II	CD30-targeting CAR T cells
NHL with CD30 expression	R/R		Phase II	BV and nivolumab combination
Hodgkin lymphoma	Treatment naïve	Advanced disease, pediatric patients	Phase II	BV plus adriamycin/inblastine/dacarbazine (AVD)
	Treatment naïve	HIV-associated HL	Phase II	BV plus adriamycin/vinblastine/dacarbazine (AVD)
	R/R		Phase I/II	BV and ifosfamide/carboplatin/etoposide (ICE)
	R/R		Phase II	BV and nivolumab combination
	R/R		Phase II	BV and ibrutinib
	R/R		Phase II	BV and everolimus
	R/R	Refractory to salvage with refractory to salvage with ifosfamide, gemcitabine, and vinolrelbine	Phase II	BV as pre-AuSCT induction
	R/R		Phase III, randomized	Pembrolizumab versus BV
ALK-positive ALCL	R/R *or* ineligible for chemotherapy		Phase II, single arm	BV and imatinib combination
CD30 positive DLBCL	R/R		Phase II, randomized	Bendamustine and rituximab (BR) versus BR with BV
	R/R		Phase II	BV and lenalidomide

Abbreviations: ALCL, anaplastic large cell lymphoma, AuSCT, autologous stem cell transplant; BV, brentuximab vedotin; CAR T cells, chimeric antigen receptor T cells; DLBCL, diffuse large B cell lymphoma; HL, Hodgkin lymphoma; NHL, non-Hodgkin lymphoma; R/R, relapsed/refractory.
